# Does the transition to grandparenthood influence the health and well-being of older people? Evidence from the CHARLS study in China

**DOI:** 10.1016/j.ssmph.2022.101328

**Published:** 2022-12-23

**Authors:** Jiawei Wu, Karen Glaser, Mauricio Avendano

**Affiliations:** a Department of Global Health & Social Medicine, King's College London, London, United Kingdom; b Institute for Social and Economic Research, University of Essex, Colchester, United Kingdom; c Center for Primary Care and Public Health (Unisanté), Department of Epidemiology and Health Systems, University of Lausanne, Lausanne, Switzerland; d Department of Social and Behavioural Sciences, Harvard School of Public Health, Boston, MA, USA

## Abstract

• Transitioning to grandmotherhood was associated with a higher probability of reporting ≥1 functional limitations in ADLs.
• Transitioning to grandparenthood was associated with higher life satisfaction.
• Role enhancement and role strain may generate mixed impacts of transitioning to grandparenthood on older adults' health.

## 1 Introduction

The transition to grandparenthood, that is, experiencing the birth of one's first grandchild, is a major event in the life of older people and their families (Taubman – Ben-Ari et al., 2018). According to generativity theory (Erikson & Erikson, 1998; Thiele & Whelan, 2008), transitioning to grandparenthood enables older adults to transmit knowledge, skills, and life experience to their grandchild, potentially bringing psychological benefits to grandparents. However, the transition to grandparenthood may also be a source of stress by reinforcing the feeling of being old and approaching death (Fung et al., 2005), changing the balance between work and family responsibilities (Lumsdaine & Vermeer, 2015), and increasing potential conflict with adult children (Goh, 2006).

The well-established role enhancement and role strain theories provide an overarching theoretical framework for understanding the potential health effects of the transition to grandparenthood. Role enhancement refers to the augmentation of a person's power, prestige, resources, and emotional gratification from occupying multiple roles (Sieber, 1974). The transition to grandparenthood expands older adults' family roles (Krause, 1988) not only because of the important role grandparents play in grandchild care, but also because it may change their self-perceptions and identity, for example, by promoting a sense of authority, meaning, and purpose (Bordone & Arpino, 2016). In contrast, role strain refers to the stress that arises from multiple demands and expectations, particularly when individuals struggle to fulfil their role obligations (Goode, 1960). Sieber (1974) differentiates two overlapping constructs of role strain: (1) role overload, and (2) role conflict. Role overload indicates that a person has limited resources (time, energy, money) to fulfil multiple role demands. For example, in many European countries, transitioning to grandparenthood predates retirement by at least five years (Leopold & Skopek, 2015). Juggling employment and potential involvement in infant support and care (e.g., financial support for new parents or temporary babysitting) may lead to role overload, particularly for grandmothers who are more affected due to the gendered nature of informal caregiving (Tanskanen et al., 2019). Role conflict arises when the agreement on the expectations of role responsibilities is not achieved between role performers and observers. Grandparents sometimes express ambivalence and contradictory attitudes towards their grandparental role due to conflicts with their adult children about babysitting expectations and child-rearing style (Goh, 2006).

### 1.1 Health effects of the transition to grandparenthood

Empirical research on the health effects of the transition to grandparenthood in the United States and Europe often finds small but beneficial effects on grandparents' psychological and mental well-being (Di Gessa et al., 2019; Kalmijn & De Graaf, 2012; Sheppard & Monden, 2019; Somary & Strieker, 1998; Tanskanen et al., 2019). However, in these settings there is a lack of research on first-time grandparents' physical functioning. Transitioning to grandparenthood also has major personal and social implications in East Asia and Southeast Asia (Mehta & Thang, 2011) where a voluminous body of literature has explored the health impact of caregiving to grandchildren under age 16. Although this strand of literature continues to grow (Silverstein & Zuo, 2021), in contrast, there is only a handful of empirical research on the health impact of the transition to grandparenthood tied to these contexts (Lai et al., 2021; Yang, 2021; Zhang et al., 2021). Similarly, these few studies focus on psychological and mental well-being outcomes, but little is known about first-time grandparents’ physical health. For example, a cross-sectional study suggests that expectant grandparents in Hong Kong are happier and report higher self-rated mental and physical health scores than those not expecting to become grandparents (Lai et al., 2021). However, expectant grandparents may not report better health after the arrival of the grandchild. Using the China Health and Retirement Longitudinal Study (CHARLS), research shows that transitioning to grandparenthood is associated with reduced depression (Yang, 2021) but has no effect on loneliness (Zhang et al., 2021).

We identified several important gaps in the literature. First, few studies have examined the impact of the transition to grandparenthood on physical health and functioning given the predominant focus on psychological and mental well-being (Di Gessa et al., 2019; Sheppard & Monden, 2019; Tanskanen et al., 2019; Yang, 2021). Second, grandparents' interaction with a new born grandchild (through co-residence and/or babysitting) is critical for understanding the impact of this transition on well-being, yet few studies have examined the birth of a first grandchild together with co-residence and grandchild care (Sheppard & Monden, 2019). The birth of a new grandchild is likely to lead to more involvement with the extended family given the infant's greater need for care and support. For example, research suggests considerable involvement of grandparents with very young grandchildren: around 35% of grandchildren aged 0–3 in the United States receive care from grandparents during at least one 3-month period (Vandell et al., 2003). Multiple surveys in China show that grandparents account for about 40% of primary at-home caregivers for children under the age of 3 (Wu & Wang, 2017). Physically demanding caregiving activities (including co-residence) may affect grandparents' physical health (Harrington Meyer & Abdul-Malak, 2020; Jendrek, 1993), as well as their psychological and mental well-being. Third, some studies have examined how the impact of transitioning to grandparenthood differs between grandfathers and grandmothers, but results seem to differ by outcome measures and are often inconclusive (Condon et al., 2018; Kalmijn & De Graaf, 2012; Sheppard & Monden, 2019; Somary & Strieker, 1998; Tanskanen et al., 2019). Last, we aim to use advanced panel data methods to better identify the effect of a first-born grandchild on older adults' health and well-being. While many studies find that people who transition to grandparenthood have better health and well-being outcomes than those who do not (Di Gessa et al., 2019; Yang, 2021), these studies are prone to bias from unmeasured confounding or reverse causality. For example, older people in worse health may be less likely to become grandparents or to provide grandparental childcare (Rutigliano, 2020), leading to spurious relationships. Likewise, unmeasured confounders such as personality traits or grandchild preferences may influence both health and the probability of having or engaging with grandchildren (Bordone & Arpino, 2021; Sheppard & Monden, 2019; Tanskanen et al., 2019). We address these methodological challenges by comparing the health and well-being of the same individual before and after having a first-born grandchild, controlling for a wide range of time-varying covariates.

### 1.2 Transitioning to grandparenthood in the Chinese context

China offers a promising setting for our research question because of the high prevalence of grandparenthood and grandparent-grandchild contact. Despite a low fertility rate, China has a low childlessness rate (Zhao et al., 2017), which means most older adults would experience the transition to grandparenthood. In addition, the female mean age at first birth is lower in China (26.6 in 2015 (Yang & Du, 2021)) than in Western European countries (which ranged from 28.4 in France to 30.8 in Italy in 2015) (Eurostat, 2022), generally resulting in an earlier transitioning to grandparenthood in China (Zhang et al., 2018). Importantly, both co-residential and non-coresidential grandparental childcare are common in China (Chen, 2014), most likely because childcare services become less available and affordable following economic reforms in recent decades (Du & Dong, 2013). In addition, high maternal labour force participation rates and limited maternity leave entitlements mean that grandparents play a critical role in the care of very young grandchildren (e.g., aged 0–3 years old) in China (Wu & Wang, 2017). As in Western countries, women are more involved than men in housekeeping and informal caregiving in China (Mann, 2000). In addition, the mandatory retirement age for the urban population is lower for women than for men (50 for blue-collar women, 55 for white-collar women, 60 for men) (Lin & Wang, 2019). As a result, it is likely that women play a more prominent role in the care of the newborn grandchild than men. Another unique dimension of China is the *hukou* (household registration) system. Introduced in the 1950s, the *hukou* system divides Chinese citizens into “agricultural (rural)” and “non-agricultural (urban)” *hukou* holders, reinforcing rural-urban socio-economic inequalities (Fan et al., 2011). In addition, economic reforms since the 1970s have given rise to significant rural-to-urban couple migration. Consequently, skipped-generation households are common in rural areas, where grandparents take on the custodial role of caring for their grandchildren (Cong & Silverstein, 2012).

Our study contributes to the literature in this area by addressing three important research questions in the context of China: First, what are the effects of the transition to grandparenthood on older people's physical, psychological and mental health and well-being? Second, is the effect of the transition to grandparenthood on health and well-being dependent on the level of involvement in caring or co-residence with the newborn grandchild? And third, does the impact of having a first-born grandchild on health and well-being differ by grandparent's gender and *hukou* status? We focus on the impact of the transition to grandparenthood for the first time, as we hypothesize that this transition leads to a major change in older people's perceptions of their role in society, their family relationships, and their social engagement. Based on role enhancement theory, we expect that the transition to grandparenthood leads to a beneficial effect on the physical as well as psychological and mental health and well-being of older adults. We expect this effect to be stronger for individuals who are involved in co-residing with or caring for their grandchildren, as they are likely to experience greater role enhancement/strain than grandparents who are less involved in the life of the newborn grandchild. We also expect the effect of transitioning to grandparenthood to vary by grandparents' gender, given women's more prominent role in caring for grandchildren. In addition, we expect the effect of transitioning to grandparenthood to differ by grandparents' *hukou* status, given rural grandparents' more intensive involvement in the custodial care of grandchildren, which may lead to role strain.

## 2 Methods

### 2.1 Data

Data come from the China Health and Retirement Longitudinal Study (CHARLS), a nationally representative study of community-dwelling adults aged 45 and above. Through a multi-stage probability-proportional-to-size (PPS) sampling technique, the final sample in the 2011 national baseline wave comprised 17,708 participants (10,069 main respondents and 7,639 spouses) followed every two years (Zhao et al., 2014). In this study, we used waves 2011, 2013 and 2015, and selected participants who were present in at least two of these waves. We excluded duplicate cases (n = 20); participants who had no living child (n = 569) at the time of first interview; and participants who were under age 45 at the time of first interview (n = 428). We excluded participants who did not have valid information on the total number of grandchildren in at least two waves (n = 1,742). These excluded participants, however, did not significantly differ from the eligible sample in terms of socio-demographic characteristics. This resulted in 15,351 participants among whom 12,406 were present at all three waves; 1,037 at waves 2011 and 2013; 667 at waves 2011 and 2015; and 1,241 at waves 2013 and 2015. Among these, our main analysis focused on a sample of 3,449 participants who did not have a grandchild in either 2011 or 2013. We focus on this particular sample as our objective was to assess changes in outcomes in response to transitioning to grandparenthood for the first time. Additional analyses on participants who already had a grandchild (n = 11,902) provided no extra insights and were not reported.

### 2.2 Measures

#### 2.2.1 Outcomes

Informed by prior studies (Di Gessa et al., 2019; Lai et al., 2021; Sheppard & Monden, 2019; Tanskanen et al., 2019; Yang, 2021), we included a wide range of physical as well as psychological and mental health and well-being outcomes: activities of daily living (ADLs), instrumental activities of daily living (IADLs), life satisfaction, and depressive symptoms.

*ADLs and IADLs*. In CHARLS questionnaire, participants' ADLs were assessed by reporting difficulty in dressing, bathing/showering, eating, getting into or out of bed, using the toilet, and controlling urination and defecation (internal reliability: Cronbach's α = 0.86 in 2011, α = 0.84 in 2013, α = 0.83 in 2015). For each of these six tasks, participants were asked to choose one of the following options: (1) no, I do not have any difficulty; (2) I have difficulty but still can do it; (3) yes, I have difficulty and need help; (4) I cannot do it. We followed He et al. (2019), assuming participants had no difficulty with this task only if they answered (1) no, I do not have any difficulty. By summarizing responses to these six tasks, we created a binary variable indicating whether participants had at least one ADL limitation. For IADLs, each participant was asked whether he or she had difficulty in doing household chores, preparing hot meals, shopping for groceries, making phone calls, taking medication and managing money (internal reliability: Cronbach's α = 0.85 in 2011, α = 0.80 in 2013, α = 0.80 in 2015). We performed the same operationalization, creating a binary variable for IADLs.

*Life satisfaction*. Participants were asked to what extent they were satisfied with their life. They were required to choose only one answer from a 5-point Likert scale: “completely satisfied, very satisfied, somewhat satisfied, not very satisfied, and not at all satisfied”. We recoded this variable so that 1 corresponded to “not satisfied at all” and 5 corresponded to “fully satisfied”.

*Depressive symptoms*. CHARLS uses the 10-item Center for Epidemiologic Studies-Depression (CES-D) scale to assess depressive symptoms (Internal reliability: Cronbach's α = 0.81 in 2011, α = 0.76 in 2013, α = 0.80 in 2015), which has been validated in China (Chen & Mui, 2014). Participants were asked questions about their feelings in the previous week, and for each question, they were asked to select only one answer from four choices: (1) rarely or none of the time (<1 day); (2) some or a little of the time (1–2 days); (3) occasionally or a moderate amount of the time (3–4 days); (4) most or all of the time (5–7 days). We recoded answers to two positively worded questions and consistently assigned values 0, 1, 2 and 3 to these answers, creating a continuous variable of depressive symptoms ranging from 0 to 30 (higher scores indicate worse outcomes). We used a cut-off point of 10 as the threshold for depressive symptomatology (Andresen et al., 1994).

#### 2.2.2 Transitioning to grandparenthood, co-residence, and caregiving

We are interested in identifying (a) whether participants transitioned to grandparenthood during the follow-up period; (b) whether participants co-resided with the new grandchild(ren); and (c) whether participants provided care for the new grandchild(ren). For (a), we inferred a transition to grandparenthood status by comparing the total number of grandchildren reported between waves. The total number of grandchildren was derived from questions on the number of children for every adult child of the participants. In 2011, the question was “How many sons does [Child's name] have?” and “How many daughters does [Child's name] have?”. In CHARLS 2013 and 2015, these two questions were combined into one, i.e., “How many children does [Child's name] have?”. Using this information, we created a binary independent variable indicating whether the participant had a new grandchild during the follow-up period. For (b), we identified co-residence by comparing the year and month of birth of grandchildren living in the household between wave t and the interview date for this household at wave t-1. For (c), we established whether participants provided care for a grandchild born between waves using information on whether participants provided care to children from each adult child, and whether each adult child had more children than he/she did at the previous wave (The original question is “Did you provide care for this adult child's child(ren)?”). However, we were not able to disentangle care exclusively dedicated to the newborn from caregiving commitments to each adult child's other child(ren). Based on this information, we constructed a three-category independent variable (no grandchild; neither provided care nor co-resided with newborn grandchild(ren); provided care and/or co-resided with newborn grandchild(ren)). We used the binary and categorical independent variables separately in our models.

#### 2.2.3 Covariates

In all models, we controlled for socio-demographic factors that may confound the association between transitioning to grandparenthood and health outcomes (Sheppard & Monden, 2019; Tanskanen et al., 2019). We adjusted for age, age squared, gender, marital status (married and living together or cohabiting, married but separated temporarily, separated or divorced, widowed, never married), *hukou* status (rural or urban), educational attainment (below elementary, elementary, middle or high school, college and above), employment/self-employment and individual annual income transformed to the log scale. Older adults' gender is time-invariant and we assumed that their education attainment did not vary. Although China's *hukou* system reforms facilitated the conversion between rural/urban *hukou* status, for practical or legal reasons a large number of citizens' *hukou* status remained unchanged (24 out of 3,449 respondents converted from rural *hukou* holders to urban *hukou* holders in our study sample). When estimating models with *hukou* interaction terms, we used respondents' *hukou* status at the first interview and assumed it to be time-invariant. We regarded rural older adults as employed if they were involved in farm work, family business or if they reported to be self-employed, while other categories (unemployment, out of the labour market) were compiled into a single group. Individual annual income included wage, pension, financial assets income, government transfer and other sources of income.

### 2.3 Analytical strategy

First, we presented descriptive statistics for older adults who did not have a grandchild at the first interview (2011 for some participants and 2013 for others) (n = 3,449). We then estimated linear regression models with fixed effects for the continuous dependent variable (i.e., life satisfaction) and linear probability models with fixed effects (LpmFE) (Beck, 2020) for the binary dependent variables (i.e., if had ≥1 ADL limitation, if had ≥1 IADL limitation, and if reached the threshold of depressive symptomatology). Fixed-effects models exploit within-individual variation (Allison, 2009) in respondents' grandparenthood status - that is, from not having any grandchildren to having one or more grandchildren - to identify the effect of transitioning to grandparenthood on health and well-being. By exploiting variation within individuals over time, fixed-effect models control for confounding by unmeasured individual time-invariant confounding factors that differ between individuals (e.g., genetic factors or family orientation) (Sheppard & Monden, 2019). In addition, we also show results from random-effects models for comparison. We examined effect modification by adding interaction terms with older adults’ gender and *hukou*. The specification of the main model is as follows.

Health_*it*_ = *μ*_*t*_ + *β*_1_Grandparenthood_*it*_ + *β*_2_x_*it*_ + *α*_*i*_ + *ε*_*it*_

Health_*it*_ is outcome (ADL/IADL limitations, life satisfaction, depression) for individual *i* at time *t*; *μ*_*t*_ controls for the effects of time; Grandparenthood_*it*_ is the regressor of interest and captures whether the participant transitioned to grandparenthood between time *t* and time *t-1*; x_*it*_ captures a vector of time-variant covariates; *α*_*i*_ controls for individual time-invariant characteristics; and *ε*_*it*_ is the error term.

We focused on older adults who did not have a grandchild at the first interview (i.e., potential first-time grandparents, n = 3,449). We did not apply sampling weights for regression models because individual longitudinal weights in 2015 were not available in CHARLS. We present results from complete case analyses (CCA), but we also used multiple imputation by chained equations (MICE) to handle missing data. As results were similar, we only reported results from CCA. All analyses were performed on Stata MP version 16.0.

## 3 Results

### 3.1 Descriptive statistics

Table 1 presents descriptive statistics for participants who did not have a grandchild at first interview (2011 for some participants and 2013 for others). At the first interview, the average age was 51 years, and 49% of participants were women. Most participants held rural *hukou* status (67%), had below-college educational attainment (91%), were married and lived together (84%), and were in employment (including wage work, farm work, family business or self-employment) (89%). At the last interview, 11% of participants reported having at least one ADL limitation, while 14% reported at least one IADL limitation. Participants’ life satisfaction score slightly increased from 3 to 3.3. Around a third of participants reported depressive symptomatology in either wave. Over the study period (2011–2015), 1,699 older adults transitioned to grandparenthood (23 of them reported having adopted/step/foster children (either co-residential or non-coresidential) at first interview, implying that the proportion of non-biological grandchildren would be very low. The number of orphan grandchildren was very small, as only 18 of first-time grandparents reported having no adult child alive at last interview). Among them, the median age of first grandparenthood was 51 years (51 for women, 52 for men; 51 for rural *hukou* holders, 53 for urban *hukou* holders).

**Table 1:** Unweighted descriptive statistics of sample (n = 3,449) at first/last interviews.

	First interview		Last interview	
	Mean/Freq.	SD/%	Mean/Freq.	SD/%
**Demographics**				
Age	50.59	5.30	54.10	5.37
Male	0.51	0.50	0.51	0.50
Urban *hukou*	0.33	0.47	0.34	0.47
Education: Below elementary (ref)	686	19.89	686	19.89
Elementary	640	18.56	640	18.56
Middle or high school	1,811	52.51	1,811	52.51
College and above	202	5.86	202	5.86
Missing	110	3.19	110	3.19
Marital Status: Married/cohabiting (ref)	2,883	83.59	2,779	80.57
Married but separated temporarily	273	6.78	262	7.60
Separated or divorced	82	1.34	65	1.88
Widowed	96	9.68	148	4.29
Never married	6	0.17	4	0.12
Missing	109	3.16	191	5.54
Unemployed/out of the labour force	0.11	0.31	0.18	0.38
Log individual annual total income	5.39	4.82	4.52	4.94
**Health and Well-being**				
≥ 1 ADL limitation	0.08	0.27	0.11	0.31
≥ 1 IADL limitation	0.11	0.31	0.14	0.35
Life satisfaction (range: 1–5)	3.00	0.70	3.33	0.78
Depressive symptoms (range: 0–30)	7.20	5.82	7.26	6.10
Depression score ≥10	0.29	0.45	0.29	0.46
**Adult children and grandchildren**				
Number of living child(ren)	1.66	0.73	1.84	0.98
Number of grandchild(ren)	0	0	0.87	1.36
Had a new grandchild	n.a.	n.a.	1,699	49.26
**Contact with the newborn**				
No grandchild at all (ref)	3,449	100	1,750	50.74
Neither care for nor co-residence with newborn grandchildren	n.a.	n.a.	844	24.47
Care provision and/or co-residence with newborn grandchildren	n.a.	n.a.	855	24.79

Notes: Data source: CHARLS waves 2011, 2013, 2015. n.a. = not applicable. SD = standard errors.

### 3.2 The health and well-being effects of transitioning to grandparenthood

Table 2 shows the associations between the transition to grandparenthood and ADL limitation, IADL limitation, life satisfaction, and depressive symptomatology for our study sample (n = 3,449). Table 2 presents the results from two separate models, one using the binary variable indicating the transition to grandparenthood (top rows), and a separate model using the three-category variable (no grandchildren; had a new grandchildren but did not provide care or co-resided; and had a new grandchildren and provided care and/or co-resided). Coefficients from random- (columns 1/3/5/7) and fixed-effects (columns 2/4/6/8) models for the same health outcomes are presented in adjacent columns. In fixed-effect models, transitioning to grandparenthood was neither associated with the probability of reporting either ADL or IADL limitations nor with depressive symptomatology for grandparents, regardless of whether participants cared for, or co-resided with, their grandchildren. Transitioning to grandparenthood led to a significant increase of 0.06 points (SE = 0.03, p < 0.05) in life satisfaction scores. This effect corresponds to a Cohen's d of 0.24, which is considered a small to medium effect size. First-time grandparents who provided care and/or co-resided with the newborn experienced an increase of 0.07 points in life satisfaction scores (SE = 0.04, p < 0.05). By contrast, transitioning to grandparenthood did not improve life satisfaction scores for grandparents who neither provided care nor co-resided with the newborn. Hausman tests did not reject the null hypothesis, suggesting that differences in coefficients between random- and fixed-effects models were not significant. However, we present coefficients from fixed-effects models because random-effects models assume that unobserved time-invariant heterogeneity is not correlated with any time-variant covariate. We used the approach proposed by Mundlak (1978) to check this assumption and found that it was violated (results not shown). We prefer fixed-effects models as our main specification because, by using fixed-effects, it is possible to control for all characteristics of individuals that may confound the relationship between becoming a grandparent and health, as long as those characteristics do not change over time. This is possible because fixed-effects models make comparisons within individuals, so that each individual serves as her or his own control. This is in contrast to random-effects models, which make comparisons both within and between individuals, and are therefore more susceptible to confounding by unmeasured individual characteristics. A drawback is that fixed-effects models reduce bias at the expense of larger standard errors, because they completely ignore between-person variability. However, between-person variability is likely to lead to confounding by unmeasured personal characteristics. By restricting analysis to within-person variability, fixed-effects models can better address confounding and are more likely to yield unbiased estimates (Allison, 2009).

**Table 2:** Transitioning to grandparenthood and limitations in ADL, IADL, life satisfaction, and depression among potential first-time grandparents (n = 3,449).

	≥1 ADL limitation		≥1 IADL limitation		Life satisfaction		Depression	
	RE(1)	FE(2)	RE(3)	FE (4)	RE (5)	FE (6)	RE (7)	FE (8)
**(binary)** Transitioning to grandparenthood (vs no grandchild)	0.01	0.01	0.00	0.00	0.06**	0.06*	0.01	0.02
	(0.01)	(0.01)	(0.09)	(0.01)	(0.02)	(0.03)	(0.01)	(0.02)
**(categorical) Caring for and co-residence with the newborn** (No grandchild = ref)								
Neither provided care nor co-resided with the newborn	0.02*	0.010	0.003	−0.00	0.06*	0.04	0.02	0.02
	(0.01)	(0.01)	(0.01)	(0.02)	(0.03)	(0.03)	(0.02)	(0.02)
Provided care and/or co-resided with the newborn	0.00	0.01	−0.00	0.01	0.07*	0.07*	−0.00	0.01
	(0.01)	(0.01)	(0.01)	(0.02)	(0.03)	(0.04)	(0.02)	(0.02)
Number of observations	8,531	8,531	8,525	8,525	7,744	7,744	7,957	7,957

Notes: Data source: CHARLS 2011, 2013, 2015. RE = random effects, FE = fixed effects. Models with binary and categorical regressors were estimated separately but the results are presented together. Beta coefficients are presented. Standard errors are in parenthesis. All models control for age, age square, sex, *hukou* status, educational level, marital status, employment status and log household annual total income. **p < 0.01, *p < 0.05, + p < 0.1.

### 3.3 Gender and *hukou* status differences

Table 3 shows the results from regression models that examined the interaction between gender and transitioning to grandparenthood (female without grandchildren are the reference group). We found no evidence of an interaction for IADL limitations, life satisfaction, and depression. However, gender modified the effects of transitioning to grandparenthood on the probability of reporting at least one ADL limitation (b for interaction term = −0.06, SE = 0.02, p < 0.001). Transitioning to grandparenthood increased the probability of reporting ADL limitations among grandmothers (b = 0.04, SE = 0.01, p < 0.01), but not among grandfathers. This effect corresponds to a Cohen's d of 0.15, which is considered a small effect size. We found that only women who cared for and/or co-resided with the newborn experienced an increase in the probability of ADL limitations as a result of experiencing a first-born grandchild (b = 0.05, SE = 0.02, p < 0.01).

**Table 3:** Transitioning to grandparenthood and limitations in ADL, IADL, life satisfaction, and depression among potential first-time grandparents (n = 3,449), interaction with older adults’ gender.

	≥1 ADL limitation		≥1 IADL limitation		Life satisfaction		Depression	
	RE(1)	FE(2)	RE(3)	FE(4)	RE(5)	FE(6)	RE(7)	FE(8)
**(binary) Transitioning to grandparenthood**								
Transitioning to grandparenthood	0.03**	0.04**	0.02	0.03+	0.06*	0.07+	0.02	0.03
	(0.01)	(0.01)	(0.02)	(0.02)	(0.03)	(0.04)	(0.02)	(0.02)
Male	−0.01		−0.01		0.02		−0.07**	
	(0.01)		(0.01)		(0.02)		(0.01)	
Transitioning to grandparenthood × Male	−0.04**	−0.06***	−0.03+	−0.05*	−0.00	−0.02	−0.02	−0.02
	(0.01)	(0.02)	(0.02)	(0.02)	(0.04)	(0.04)	(0.02)	(0.03)
**(categorical) Caring & co-residing** No grandchildren (ref)								
Neither cared for nor co-resided with the newborn	0.04**	0.03	0.02	0.01	0.05	0.05	0.03	0.02
	(0.01)	(0.02)	(0.02)	(0.02)	(0.04)	(0.05)	(0.02)	(0.03)
Provided care and/or co-resided with the newborn	0.03*	0.05**	0.01	0.04*	0.08*	0.08+	0.00	0.03
	(0.01)	(0.02)	(0.02)	(0.02)	(0.04)	(0.05)	(0.02)	(0.03)
Neither cared for nor co-resided with the newborn × Male	−0.03	−0.03	−0.04+	−0.03	0.02	−0.02	−0.03	−0.00
	(0.02)	(0.02)	(0.02)	(0.03)	(0.05)	(0.06)	(0.03)	(0.03)
Provided care and/or co-resided with the newborn × Male	−0.06**	−0.09***	−0.02	−0.07*	−0.03	−0.02	−0.01	−0.04
	(0.02)	(0.02)	(0.02)	(0.03)	(0.05)	(0.06)	(0.03)	(0.04)
Number of observations	8,531	8,531	8,525	8,525	7,744	7,744	7,957	7,957

Notes: Data source: CHARLS 2011, 2013, 2015. RE = random effects; FE = fixed effects. Models with binary and categorical regressors were estimated separately but the results are presented together. Beta coefficients are presented. Standard errors are in parenthesis. All models control for age, age square, sex, *hukou* status, educational level, marital status, employment status and log individual annual total income. **p < 0.01, *p < 0.05, + p < 0.1.

Table 4 shows coefficients from regression models that examined the interaction between transitioning to grandparenthood and *hukou* status (rural *hukou* holders with no grandchildren are the reference group). *Hukou* status did not modify the impact of transitioning to grandparenthood on any of the four outcomes examined. The increase in life satisfaction after the arrival of a grandchild was stronger among rural grandparents who cared for and/or co-resided with the newborn (b = 0.10, SE = 0.04, p < 0.05), but this effect was not significant (b for interaction term = −0.12, SE = 0.07, p < 0.1).

**Table 4:** Transitioning to grandparenthood and limitations in ADL, IADL, life satisfaction, and depression among potential first-time grandparents (n = 3,449), interaction with older adults’ *hukou* status.

	≥1 ADL limitation		≥1 IADL limitation		Life satisfaction		Depression	
	RE(1)	FE(2)	RE(3)	FE(4)	RE(5)	FE(6)	RE(7)	FE(8)
(binary) Transitioning to grandparenthood								
Transitioning to grandparenthood	0.01	0.01	0.01	0.01	0.08**	0.06*	−0.00	0.02
	(0.01)	(0.01)	(0.01)	(0.01)	(0.02)	(0.03)	(0.01)	(0.02)
Urban *hukou*	−0.01	0.01	0.00	0.14*	0.08**	0.14	−0.06**	0.04
	(0.01)	(0.06)	(0.01)	(0.07)	(0.03)	(0.15)	(0.02)	(0.09)
Transitioning to grandparenthood × Urban *hukou*	−0.01	−0.02	−0.02	−0.03	−0.06	−0.04	0.03	0.01
	(0.02)	(0.02)	(0.02)	(0.02)	(0.04)	(0.05)	(0.03)	(0.03)
**(categorical) Caring & co-residing**								
No grandchild (ref)								
Neither provided care nor co-resided with the newborn	0.02+	0.01	0.01	0.00	0.06*	0.03	0.01	0.02
	(0.01)	(0.01)	(0.01)	(0.02)	(0.03)	(0.04)	(0.02)	(0.02)
Provided care and/or co-resided with the newborn	0.01	0.02	0.00	0.02	0.09**	0.10*	−0.01	0.01
	(0.01)	(0.01)	(0.01)	(0.02)	(0.03)	(0.04)	(0.02)	(0.02)
Neither provided care nor co-resided with the newborn × Urban *hukou*	−0.01	−0.02	−0.02	−0.07	−0.09	0.04	0.04	0.02
	(0.02)	(0.03)	(0.02)	(0.03)	(0.06)	(0.07)	(0.03)	(0.04)
Provided care and/or co-resided with the newborn × Urban *hukou*	−0.01	−0.02	−0.02	−0.05	−0.10+	−0.12+	0.02	−0.00
	(0.02)	(0.03)	(0.02)	(0.03)	(0.06)	(0.07)	(0.03)	(0.04)
Number of observations	8,531	8,531	8,525	8,525	7,744	7,744	7,957	7,957

Notes: Data source: CHARLS 2011, 2013, 2015. RE = random effects; FE = fixed effects. Models with binary and categorical regressors were estimated separately but the results are presented together. Beta coefficients are presented. Standard errors are in parenthesis. All models control for age, age square, sex, *hukou* status, educational level, marital status, employment status and log individual annual total income. **p < 0.01, *p < 0.05, + p < 0.1.

### 3.4 Sensitivity analyses

We conducted several sensitivity analyses to check the robustness of our key findings in Table 2, Table 3 We estimated models that excluded 18 respondents who reported having no adult child alive at last interview despite having grandchildren (i.e., these grandchildren were orphan grandchildren). Results were nearly identical to those for the full sample (results available upon request). We also estimated models that excluded 23 respondents who reported having adopted, step or foster children (co-residential or non-coresidential) at baseline to exclude non-biological grandchildren. Results were nearly identical to those for the full sample (results available upon request).

We alternatively estimated logit models with fixed effects (“LogitFE”) (Beck, 2020) for binary health outcomes. The findings (null effect in Table A1 and significant effects on ADL limitation in Table A2) were similar to those shown in Table 2, Table 3. We followed Beck (2020) and estimated LpmFE dropping all-zero groups, and obtained very similar results as those observed in Table 3 (results not shown).

We also estimated OLS models using pooled sample and compared the results (presented in Tables A3, A4, and A5) with our main findings (Table 2, Table 3, Table 4) from fixed-effects models. In OLS models, the transition to grandparenthood was associated with a 0.18 increase in life satisfaction scores (Table A3), an effect that was three times larger than the effect observed in fixed-effects models (Table 2). In both OLS (Table A4) and fixed effect models (Table 3), we found evidence of a gender interaction for the probability of having ≥1 ADL limitation, but the magnitude of the interaction was half smaller in OLS models (Table A4). Overall, these results suggest that at least part of the association between becoming a grandparent and health or life satisfaction is potentially due to unobserved differences between individuals, which we try to control for using fixedeffects models.

To explore differences between co-residing and non-coresidential first-time grandparents, we further broke down the sample into four categories of grandparents who: (a) had a new grandchild but did not provide care or co-resided with grandchildren; (b) had a new grandchild and provided care but did not co-reside; (c) had a new grandchild and co-resided with him/her, but did not provide care; (d) had a new grandchild, provided care and co-resided. We also provide some descriptive statistics of the prevalence of these categories (Table A6) and re-estimated regression models (Tables A7 and A8). As suggested by these descriptive statistics, among first-time grandparents, half of them provided care to the newborn but did not co-reside. In addition, only 0.67% of first-time grandparents co-resided but did not provide care, suggesting that most co-residing grandparents were providing some grandchild care. Interestingly, the positive effect of providing care for grandchildren on life satisfaction (Table 2) was not different between grandparents who co-resided and those who did not co-reside (although not statistically significant for the latter group possibly due to much smaller sample size) with their grandchildren (Table A7). The negative effect of providing care for grandchildren on first-time grandmothers’ ADLs (Table 3) was stronger for first-time grandmothers who co-resided than for those who did not co-reside with their grandchildren (rows for main effects in Table A8).

## 4 Discussion

In this study, we explored the physical, psychological, and mental health and well-being effects of transitioning to grandparenthood and considered intergenerational contact with the newborn (caregiving and/or co-residence). We drew on nationally representative longitudinal data from CHARLS and estimated fixed-effects models which handle selection bias by exploiting within-individual variation only. Overall, our results do not show an impact of first-time grandparenthood on physical functioning or depressive symptoms in the study sample. However, we did find that transitioning to grandmotherhood was associated with a higher probability of reporting ADL limitations, a finding driven by a negative impact on first-time grandmothers who care for and/or co-reside with the newborn. Our findings also suggest that transitioning to grandparenthood was associated with higher life satisfaction scores among first-time grandparents, particularly for those who care for and/or co-reside with newborns.

The association between transitioning to grandmotherhood and a higher probability of reporting ADL limitations concentrated on first-time grandmothers who cared for and/or co-resided with their newborn grandchild(ren). These findings are consistent with role strain theory, suggesting that multiple roles can lead to physical exhaustion and accumulated stress. Role accumulation may lead to “wear and tear” of bodily systems, potentially increasing chronic stress and worsening physical health. Social exchange theory predicts that grandparental stress arises when the demands of caring for grandchildren exceeds the resources available to grandparents to succeed in their new role (Hayslip & Fruhauf, 2019). Raising newborns may lead to an accelerated decline of physical functioning, which may put grandparents at risk of physical health problems. Fixed-effect models include time fixed effects, which capture the impact of time-changing factors that were shared across individuals, e.g., ageing. However, there may still be changes correlated with becoming a grandparent that we did not measure and that may bias our results. In addition, the finding that an adverse impact of becoming a grandparent on ADLs was only found for women may be due to heterogeneous effects by health status, i.e., older women have worse physical functioning and more disability than men (Wheaton & Crimmins, 2016).

Echoing previous findings (Di Gessa et al., 2019; Lai et al., 2021; Somary & Strieker, 1998; Tanskanen et al., 2019), the positive association with life satisfaction is consistent with role enhancement theory (Sieber, 1974). Transitioning to grandparenthood may expand older adults’ family roles (Krause, 1988), conferring a new identity and enhancing their prestige. Grandparents may be more satisfied with life because the arrival of the grandchild enhances their status within the household. Our finding that grandparents who care for and/or co-reside with the newborn experience increased life satisfaction is in line with generativity theory (Erikson & Erikson, 1998), which predicts that in establishing and guiding future generations grandparents derive psychological well-being associated with babysitting for their offspring.

In contrast to our original hypothesis, we find no evidence that the impact of transitioning to grandparenthood differed according to *hukou* status. This suggests that both urban and rural older adults experience similar benefits and risk when entering grandparenthood. Given the general lack of formal childcare services in China, both rural and urban grandparents –particularly women-are likely to be involved in caring and/or co-reside with the young offspring (Du & Dong, 2013; Mann, 2000; Wu & Wang, 2017). Yet, the experience of grandparenthood is likely to differ for rural and urban residents, as each group experiences different kinds of challenges. Urban blue-collar grandmothers are exposed to physically demanding work environments and may experience physical health deterioration throughout their employment histories and beyond retirement (Schreuder et al., 2008). By contrast, Song and Smith (2019) find that rural *hukou* adults living in rural areas face challenges associated with limited access to healthcare, which places them at risk of physical health decline. Further research is required to examine in more detail potential differences in the experience of grandparenting between urban and rural *hukou* holders.

Our study highlights the importance of considering the new questions that arise in the face of a growing number of intergenerational households, partly because of increased longevity of older people and their enhanced role in caring for grandchildren. Silverstein et al. (2006) noted the psychological benefits of downward intergenerational transfers (e.g., grandparental childcare) for older adults in multigenerational households in rural China. Our results highlight the fact that becoming a grandparent in the context of co-residence with grandchildren may involve demanding caregiving activities that may affect grandparents’ health, consistent with earlier studies (Harrington Meyer & Abdul-Malak, 2020; Jendrek, 1993).

Overall, we contribute to the literature by showing the detrimental effects on first-time grandmothers' ADLs. Our finding of small beneficial effects on first-time grandparents’ psychological and mental well-being is consistent with previous studies using fixed-effects models (Sheppard & Monden, 2019; Tanskanen et al., 2019). The null finding on depression in our study, in contrast to prior research (Yang, 2021), may suggest that previously observed effects do not hold after confounding by time-invariant characteristics or between-individual variation is controlled for in fixed-effects models (Bordone & Arpino, 2021). Our results therefore adds robust evidence to the literature on the health and well-being effects of transitioning to grandparenthood.

Some important limitations should be considered in our study. First, we were not able to ascertain whether changes in the provision of care to grandchildren was due to the arrival of a new grandchild, or to other family changes that may have occurred in families that had a new grandchild. Neither can we rule out the possibility of misclassification of co-residence, as some newborns may have lived in the household but moved out between interviews. Nevertheless, we do not expect these two issues to substantially bias our estimates or change the overall pattern of results.

Second, previous studies showed that lineage modified the effects of transitioning to grandparenthood (Di Gessa et al., 2019; Somary & Strieker, 1998). We only considered grandparents' gender as CHARLS provides information on grandchildren's gender only if they live in the same household. Although traditional patrilineality may have weakened (Zhang et al., 2019), paternal grandparents may still be more affected by transitioning to grandparenthood than maternal grandparents. Future work should explore this question using more detailed data on offspring's gender.

Third, the transiency (less than 16 months) of the effects of transitioning to grandparenthood was cautioned by Di Gessa et al. (2019). Moreover, expecting the birth of a future grandchild during interview may already bring health benefits to older adults before the new birth (Di Gessa et al., 2019). CHARLS only records the precise year and month of birth of grandchildren if they live in the household, so we were unable to estimate the time lag between the birth and interview dates. A strength of our study is that we are able to capture the impact of becoming a grandparent on health and well-being up to 4 years after the newborn's arrival. This distinguishes our analysis from that in other papers that examine differences between grandparents with grandchildren below the age of 16 and older people without grandchildren below this age. However, we were not able to assess whether the impact of becoming a grandparent may change over time, as people grow older and the meaning of the grandparent role evolves (Silverstein & Marenco, 2001). Future studies with more detailed data on older people's time use (Di Gessa et al., 2020) may be required to capture how grandparents' role changes over time, and identify the implications for the their health and well-being.

## 5 Conclusion and implications

Our study contributes to the literature by showing that elements from both role enhancement and role strain theories may be important in understanding the impact of transitioning to grandparenthood on grandparents' physical, psychological, and mental health and well-being. We found harmful effects for first-time grandmothers' ADL limitations but beneficial effects for first-time grandparents' life satisfaction. A possible implication is that policies that encourage the participation of older people in the care of very young grandchildren are likely to have mixed effects: increasing life satisfaction, but also placing some grandparents – particularly women who provide care for young grandchildren – at increased risk of faster functional decline. A potential alternative may be to combine policies that promote grandparent's participation in raising grandchildren (e.g., cash for care programmes) with policies that mitigate the risk that grandparents become the main source of childcare for working parents. For example, Lin and Wang (2019) find that the introduction of childcare centres targeting children aged 3-5 decreased grandmothers' reports of chronic disease and depression. In 2019, China's State Council announced an initiative to improve formal childcare services for children aged 0–3. Such policies may enable grandparents to be involved in the care and rearing of grandchildren, while offering working parents alternative sources of childcare, with potentially beneficial consequences for the well-being of grandparents, parents and grandchildren. Due to ongoing changes in marriage and family (Raymo et al., 2015), the transition to grandparenthood in China may become less common (due to increasing childlessness rates among adult children) and at later ages (due to delayed marriage and the transition to parenthood). Future research is needed to investigate the evolving nature of the transition to grandparenthood in China and its impact on older adults' well-being.

## Author agreement

All authors have seen and approved the final version of the manuscript being submitted.

## Funding source declaration

This work was supported by a PhD scholarship from the 10.13039/501100004543China Scholarship Council. This paper represents independent research partly supported by the 10.13039/501100000269Economic and Social Research Council Centre for Society and Mental Health at 10.13039/501100000764King's College London (Grant Reference: ES/S012567/1). Additional support was received from an UK Economic and Social Research Council grant “Families, households and health in ageing populations: Projections and implications” (Grant Reference: ES/T014083/1).

## Permission note

This paper performed secondary data analysis on survey data which have obtained ethical approval before being fielded and are publicly accessible.

## CRediT authorship contribution statement

**Jiawei Wu:** Conceptualization, Data curation, Formal analysis, Writing – original draft, Funding acquisition. **Karen Glaser:** Conceptualization, Writing – review & editing. **Mauricio Avendano:** Conceptualization, Writing – review & editing, Funding acquisition.

## Declaration of competing interest

None.

## Data Availability

Data will be made available on request.

## References

[bib1] Allison P.D. (2009). Fixed effects regression models.

[bib2] Andresen E.M., Malmgren J.A., Carter W.B., Patrick D.L. (1994). Screening for depression in well older adults: Evaluation of a short form of the CES-D. American Journal of Preventive Medicine.

[bib3] Beck N. (2020). Estimating grouped data models with a binary-dependent variable and fixed effects via a logit versus a linear probability model: The impact of dropped units. Political Analysis.

[bib4] Bordone V., Arpino B. (2016). Do grandchildren influence how old you feel?. Journal of Aging and Health.

[bib5] Bordone V., Arpino B. (2021). Is there a rejuvenating effect of (Grand)Childcare? A longitudinal study on German data. The Journals of Gerontology: Serie Bibliographique.

[bib6] Chen F., Poston J.D.L., Yang W.S., Farris D.N. (2014). The family and social change in Chinese societies.

[bib7] Chen H., Mui A.C. (2014). Factorial validity of the center for epidemiologic studies depression scale short form in older population in China. International Psychogeriatrics.

[bib8] Condon J., Luszcz M., McKee I. (2018). The transition to grandparenthood: A prospective study of mental health implications. Aging & Mental Health.

[bib9] Cong Z., Silverstein M., Mehta K.K., Thang L.L. (2012). Experiencing grandparenthood: An asian perspective.

[bib10] Di Gessa G., Bordone V., Arpino B. (2019). Becoming a grandparent and its effect on well-being: The role of order of transitions, time, and gender. The Journals of Gerontology: Serie Bibliographique.

[bib11] Di Gessa G., Zaninotto P., Glaser K. (2020). Looking after grandchildren: Gender differences in ‘when,’ ‘what,’ and ‘why’: Evidence from the English longitudinal study of ageing. Demographic Research.

[bib12] Du F., Dong X.-y. (2013). Women's employment and child care choices in urban China during the economic transition. Economic Development and Cultural Change.

[bib13] Erikson E.H., Erikson J.M. (1998).

[bib14] Eurostat (2022). https://ec.europa.eu/eurostat/databrowser/view/DEMO_FIND/bookmark/table?lang=en&bookmarkId=26565240-1494-4163-bf9c-4bb7215590ff.

[bib15] Fan C.C., Sun M., Zheng S. (2011). Migration and split households: A comparison of sole, couple, and family migrants in Beijing, China. Environment and Planning A: Economy and Space.

[bib16] Fung H.H., Siu C.M.Y., Choy W.C.W., McBride-Chang C. (2005). Meaning of grandparenthood: Do concerns about time and mortality matter?. Ageing International.

[bib17] Goh E.C.L. (2006). Raising the precious single child in urban China-an intergenerational joint mission between parents and grandparents. Journal of Intergenerational Relationships.

[bib18] Goode W.J. (1960). A theory of role strain. American Sociological Review.

[bib19] Harrington Meyer M., Abdul-Malak Y., Harrington Meyer M., Abdul-Malak Y. (2020). Grandparenting children with disabilities.

[bib20] Hayslip B.J., Fruhauf C.A. (2019). Grandparenting: Influences on the dynamics of family relationships.

[bib21] He M., Ma J., Ren Z., Zhou G., Gong P., Liu M., Yang X., Xiong W., Wang Q., Liu H., Zhang X. (2019). Association between activities of daily living disability and depression symptoms of middle-aged and older Chinese adults and their spouses: A community based study. Journal of Affective Disorders.

[bib22] Jendrek M.P. (1993). Grandparents who parent their grandchildren: Effects on lifestyle. Journal of Marriage and Family.

[bib23] Kalmijn M., De Graaf P.M. (2012). Life course changes of children and well-being of parents. Journal of Marriage and Family.

[bib24] Krause N. (1988). Positive life events and depressive symptoms in older adults. Behavioral Medicine.

[bib25] Lai D.W.L., Li J., Bai X. (2021). To be or not to be: Relationship between grandparent status and health and wellbeing. BMC Geriatrics.

[bib26] Leopold T., Skopek J. (2015). The demography of grandparenthood: An international profile. Social Forces.

[bib27] Lin M., Wang Q. (2019). Center-based childcare expansion and grandparents' employment and well-being. Social Science & Medicine.

[bib28] Lumsdaine R.L., Vermeer S.J.C. (2015). Retirement timing of women and the role of care responsibilities for grandchildren. Demography.

[bib29] Mann S., Entwisle B., Henderson G.E. (2000). Re-Drawing boundaries: Work, household, and gender in China.

[bib30] Mehta K.K., Thang L.L. (2011).

[bib31] Mundlak Y. (1978). On the pooling of time series and cross section data. Econometrica.

[bib32] Raymo J.M., Park H., Xie Y., Yeung W.-j. J. (2015). Marriage and family in East Asia: Continuity and change. Annual Review of Sociology.

[bib33] Rutigliano R. (2020). Counting on potential grandparents? Adult children's entry into parenthood across European countries. Demography.

[bib34] Schreuder K.J., Roelen C.A., Koopmans P.C., Groothoff J.W. (2008). Job demands and health complaints in white and blue collar workers. Work.

[bib35] Sheppard P., Monden C. (2019). Becoming a first-time grandparent and subjective well-being: A fixed effects approach. Journal of Marriage and Family.

[bib36] Sieber S.D. (1974). Toward a theory of role accumulation. American Sociological Review.

[bib37] Silverstein M., Cong Z., Li S. (2006). Intergenerational transfers and living arrangements of older people in rural China: Consequences for psychological well-being. The Journals of Gerontology: Serie Bibliographique.

[bib38] Silverstein M., Marenco A. (2001). How Americans enact the grandparent role across the family life course. Journal of Family Issues.

[bib39] Silverstein M., Zuo D. (2021). Grandparents caring for grandchildren in rural China: Consequences for emotional and cognitive health in later life. Aging & Mental Health.

[bib40] Somary K., Strieker G. (1998). Becoming a grandparent: A longitudinal study of expectations and early experiences as a function of sex and lineage. The Gerontologist.

[bib41] Song Q., Smith J.P. (2019). Hukou system, mechanisms, and health stratification across the life course in rural and urban China. Health & Place.

[bib42] Tanskanen A.O., Danielsbacka M., Coall D.A., Jokela M. (2019). Transition to grandparenthood and subjective well-being in older Europeans: A within-person investigation using longitudinal data. Evolutionary Psychology.

[bib43] Taubman – Ben-Ari O., Ben Shlomo S., Findler L., Demir M., Sümer N. (2018). Close relationships and happiness across cultures.

[bib44] Thiele D.M., Whelan T.A. (2008). The relationship between grandparent satisfaction, meaning, and generativity. The International Journal of Aging and Human Development.

[bib45] Vandell D.L., McCartney K., Owen M.T., Booth C., Clarke-Stewart A. (2003). Variations in child care by grandparents during the first three years. Journal of Marriage and Family.

[bib46] Wheaton F.V., Crimmins E.M. (2016). Female disability disadvantage: A global perspective on sex differences in physical function and disability. Ageing and Society.

[bib47] Wu F., Wang L. (2017). Zhongguo xuelingqian ertong jiating zhaoliao anpai yu zhengce xuqiu — jiyu duoyuan shuju de fenxi [Family care arrangements and policy needs of preschool children in China: An analysis based on multiple data sources]. Ren Kou Yan Jiu.

[bib48] Yang Y. (2021). A multilevel analysis of the impact of transitioning to grandparenthood on individuals' depression in England, Europe and China. Aging & Mental Health.

[bib49] Yang J., Du S. (2021). Family change in China: a-70 year perspective. China Population and Development Studies.

[bib50] Zhang J., Emery T., Dykstra P. (2018). Grandparenthood in China and western Europe: An analysis of CHARLS and SHARE. Advances in Life Course Research.

[bib51] Zhang J., Fokkema T., Arpino B. (2021). Loneliness among Chinese older adults: The role of grandparenthood and grandparental childcare by gender. Journal of Family Issues.

[bib52] Zhang C., Fong V.L., Yoshikawa H., Way N., Chen X., Lu Z. (2019). The rise of maternal grandmother child care in urban Chinese families. Journal of Marriage and Family.

[bib53] Zhao Y., Hu Y., Smith J.P., Strauss J., Yang G. (2014). Cohort profile: The China health and retirement longitudinal study (CHARLS). International Journal of Epidemiology.

[bib54] Zhao Z., Xu Q., Yuan X. (2017). Far below replacement fertility in urban China. Journal of Biosocial Science.

