# Supplementary material for: Does the transition to grandparenthood influence the health and well-being of older people? Evidence from the CHARLS study in China
Source: SSM Popul Health. 2022 Dec 23;21:101328. doi: 10.1016/j.ssmph.2022.101328 (PMC9813572; doi:10.1016/j.ssmph.2022.101328)
Supplement: Multimedia component 1 [file mmc1.docx]

**Appendices for manuscript** “Does the transition to grandparenthood influence the health and well-being of older people? Evidence from the CHARLS study in China”

Table A1. The association between transitioning to grandparenthood and limitations in ADL, IADL and depression on potential first-time grandparents (n=3,449), LogitFE

|  | ≥ 1 ADL limitation | | ≥ 1 IADL limitation | | Depression | |
| --- | --- | --- | --- | --- | --- | --- |
|  | RE | FE | RE | FE | RE | FE |
| **Potential first-time grandparents** |  |  |  |  |  |  |
| **(binary)** Whether had a grandchild (vs no grandchild) | 0.16  (0.13) | 0.14  (0.18) | -0.02  (0.11) | 0.01  (0.15) | 0.05  (0.10) | 0.12  (0.12) |
| **(categorical)** No grandchild (ref) |  |  |  |  |  |  |
| Neither provided care nor co-resided with the new-born | 0.24  (0.16) | 0.13  (0.22) | -0.00  (0.13) | -0.06  (0.18) | 0.13  (0.12) | 0.14  (0.15) |
| Provided care and/or co-resided with the new-born | 0.06  (0.17) | 0.16  (0.23) | -0.04  (0.14) | 0.09  (0.19) | -0.03  (0.12) | 0.09  (0.16) |
| Number of observations | 8,531 | 1,378 | 8,525 | 1,866 | 7,957 | 2,737 |

Notes: Data source: CHARLS 2011, 2013, 2015. Beta coefficients for depression, limitations in ADL, IADL are presented. Standard errors are in parenthesis. Socio-demographic characteristics and year dummies were controlled for.

Table A2. The association between transitioning to grandparenthood and limitations in ADL, IADL, and depression on potential first-time grandparents (n=3,449), **with a gender interaction term**, LogitFE

|  | ≥ 1 ADL limitation | | ≥ 1 IADL limitation | | Depression | |
| --- | --- | --- | --- | --- | --- | --- |
|  | RE | FE | RE | FE | RE | FE |
| **(binary)** Whether had a grandchild (vs no grandchild) | 0.38*  (0.16) | **0.45***  (0.22) | 0.08  (0.14) | 0.17  (0.18) | 0.10  (0.12) | 0.18  (0.15) |
| Male | -0.18 |  | -0.16 |  | -0.51** |  |
|  | (0.15) |  | (0.12) |  | (0.11) |  |
| Whether had a grandchild | -0.52* | **-0.70*** | -0.23 | -0.39+ | -0.11 | -0.14 |
| × Male | (0.22) | (0.28) | (0.18) | (0.23) | (0.16) | (0.19) |
| **(categorical)** No grandchild (ref) |  |  |  |  |  |  |
| Neither cared nor co-resided with the new-born | 0.37+  (0.20) | 0.19  (0.27) | 0.12  (0.17) | 0.01  (0.22) | 0.20  (0.15) | 0.14  (0.19) |
| Cared and/or co-resided with the new-born | 0.39+  (0.21) | **0.80***  (0.32) | 0.04  (0.17) | 0.34  (0.23) | 0.00  (0.15) | 0.22  (0.20) |
| Male | -0.18 |  | -0.16 |  | -0.51** |  |
|  | (0.15) |  | (0.12) |  | (0.11) |  |
| Neither cared nor co-resided  with the new-born  × Male | -0.29  (0.28) | -0.13  (0.37) | -0.27  (0.23) | -0.17  (0.31) | -0.16  (0.21) | -0.01  (0.26) |
| Provided care and/or co-resided with the new-born × Male | -0.81**  (0.31) | **-1.38****  (0.41) | -0.19  (0.25) | -0.64+  (0.33) | -0.06  (0.21) | -0.29  (0.27) |
| Number of observations | 8,531 | 1,378 | 8,525 | 1,866 | 7,957 | 2,737 |

Notes: Data source: CHARLS 2011, 2013, 2015. RE = random effects; FE = fixed effects. Beta coefficients are presented. Standard errors are in parenthesis. Socio-demographic characteristics and year dummies were controlled for. All estimations were performed on complete cases.

** p<0.01, * p<0.05, + p<0.1

Table A3. Transitioning to grandparenthood and limitations in ADL, IADL, life satisfaction, and depression among potential first-time grandparents (n=3,449), OLS models

|  | ≥ 1 ADL limitation | ≥ 1 IADL limitation | Life satisfaction | Depression | |  |
| --- | --- | --- | --- | --- | --- | --- |
| **(binary)** Transitioning to grandparenthood (vs no grandchild) | 0.01 | 0.00 | 0.18*** | | 0.00 | |
|  | (0.01) | (0.01) | (0.02) | | (0.01) | |
| **(categorical) Caring and co-residence** **with the new-born** (No grandchild = ref) |  |  |  | |  | |
| Neither provided care nor co-resided with the new-born | 0.02** | 0.01 | 0.17*** | | 0.01 | |
|  | (0.01) | (0.01) | (0.03) | | (0.02) | |
| Provided care and/or co-resided with the new-born | 0.00 | 0.00 | 0.18*** | | -0.01 | |
|  | (0.01) | (0.01) | (0.03) | | (0.01) | |
| Number of observations | 8,531 | 8,525 | 7,744 | | 7,957 | |

Notes: Data source: CHARLS 2011, 2013, 2015. Models with binary and categorical regressors were estimated separately but the results are presented together. Beta coefficients are presented. Standard errors are in parenthesis. All models control for age, age square, sex, *hukou* status, educational level, marital status, employment status and log household annual total income. *** p<0.001, **p<0.01

Table A4. Transitioning to grandparenthood and limitations in ADL, IADL, life satisfaction, and depression among potential first-time grandparents (n=3,449), OLS models, interaction with older adults’ gender

|  | ≥ 1 ADL limitation | ≥ 1 IADL limitation | Life satisfaction | Depression | |  |
| --- | --- | --- | --- | --- | --- | --- |
| **(binary)** Transitioning to grandparenthood (vs no grandchild) |  |  |  | |  | |
| Transitioning to grandparenthood | 0.03** | 0.02 | 0.17*** | | 0.01 | |
|  | (0.01) | (0.01) | (0.03) | | (0.02) | |
| Male | -0.01 | -0.01 | 0.01 | | -0.06*** | |
|  | (0.01) | (0.01) | (0.02) | | (0.01) | |
| Transitioning to grandparenthood × Male | -0.03* | -0.02 | 0.01 | | -0.02 | |
|  | (0.01) | (0.02) | (0.04) | | (0.02) | |
| **(categorical) Caring and co-residence** **with the new-born** (No grandchild = ref) |  |  |  | |  | |
| Neither provided care nor co-resided with the new-born | 0.04** | 0.03* | 0.15*** | | 0.03 | |
|  | (0.01) | (0.01) | (.04) | | (0.02) | |
| Provided care and/or co-resided with the new-born | 0.02 | 0.00 | 0.19*** | | -0.01 | |
|  | (0.01) | (0.01) | (0.03) | | (0.02) | |
| Neither provided care nor co-resided with the new-born  × Male | -0.03 | -0.04* | 0.04 | | -0.04 | |
|  | (0.02) | (0.02) | (0.05) | | (0.03) | |
| Provided care and/or co-resided with the new-born  × Male | -0.04* | -0.01 | -0.03 | | -0.01 | |
|  | (0.02) | (0.02) | (0.05) | | (0.03) | |
| Number of observations | 8,531 | 8,525 | 7,744 | | 7,957 | |

Notes: Data source: CHARLS 2011, 2013, 2015. Models with binary and categorical regressors were estimated separately but the results are presented together. Beta coefficients are presented. Standard errors are in parenthesis. All models control for age, age square, *hukou* status, educational level, marital status, employment status and log household annual total income. *** p<0.001, **p<0.01, P<0.05

Table A5. Transitioning to grandparenthood and limitations in ADL, IADL, life satisfaction, and depression among potential first-time grandparents (n=3,449), OLS models, interaction with older adults’ *hukou* status

|  | ≥ 1 ADL limitation | ≥ 1 IADL limitation | Life satisfaction | Depression | |  |
| --- | --- | --- | --- | --- | --- | --- |
| **(binary)** Transitioning to grandparenthood (vs no grandchild) |  |  |  | |  | |
| Transitioning to grandparenthood | 0.01 | 0.01 | 0.20*** | | -0.01 | |
|  | (0.01) | (0.01) | (0.02) | | (0.01) | |
| Urban *hukou* | -0.01 | 0.00 | 0.07** | | -0.06*** | |
|  | (0.01) | (0.01) | (0.03) | | (0.01) | |
| Transitioning to grandparenthood  × Urban *hukou* | 0.00 | -0.02 | -0.08 | | .04 | |
|  | (0.02) | (0.02) | (0.04) | | (0.03) | |
| **(categorical) Caring and co-residence** **with the new-born** (No grandchild = ref) |  |  |  | |  | |
| Neither provided care nor co-resided with the new-born | 0.03* | 0.02 | 0.18*** | | 0.00 | |
|  | (0.01) | (0.01) | (0.03) | | (0.02) | |
| Provided care and/or co-resided with the new-born | 0.00 | 0.00 | 0.21*** | | -0.02 | |
|  | (0.01) | (0.01) | (0.03) | | (0.02) | |
| Neither provided care nor co-resided with the new-born  × Urban *hukou* | 0.00 | -0.03 | -0.04 | | 0.05 | |
|  | (0.02) | (0.02) | (0.06) | | (0.04) | |
| Provided care and/or co-resided with the new-born  × Urban *hukou* | -0.01 | -0.01 | -0.11 | | 0.04 | |
|  | (0.02) | (0.02) | (0.06) | | (0.03) | |
| Number of observations | 8,531 | 8,525 | 7,744 | | 7,957 | |

Notes: Data source: CHARLS 2011, 2013, 2015. Models with binary and categorical regressors were estimated separately but the results are presented together. Beta coefficients are presented. Standard errors are in parenthesis. All models control for age, age square, gender, educational level, marital status, employment status and log household annual total income. ***p<0.001, **p<0.01, *p<0.05, +p<0.1

Table A6. Additional unweighted descriptive statistics of sample’s (n = 3,449) contact with the new-born grandchild(ren) at first/last interviews

| Contact with the new-born | Fist interview | | Last interview | |
| --- | --- | --- | --- | --- |
| No grandchild at all (ref) | 3,449 | 100% | 1,750 | 50.74% |
| Had new grandchild(ren), neither provided care nor co-resided | n.a. | n.a. | 844 | 24.47% |
| Had new grandchild(ren), provided care, did not co-reside | n.a. | n.a. | 740 | 21.46% |
| Had new grandchild(ren), did not provide care, co-resided | n.a. | n.a. | 23 | 0.67% |
| Had new grandchild(ren), provided care, co-resided | n.a. | n.a. | 92 | 2.67% |

Notes: Data source: CHARLS waves 2011, 2013, 2015. n.a. = not applicable

Table A7. Transitioning to grandparenthood and life satisfaction among potential first-time grandparents (n=3,449) using a detailed category of grandparent-grandchild contact, fixed-effects models

| Contact with the new-born | Life satisfaction |
| --- | --- |
| No grandchild at all (ref) |  |
| Had new grandchild(ren), neither provided care nor co-resided | 0.04 (0.03) |
| Had new grandchild(ren), provided care, did not co-reside | 0.07* (0.04) |
| Had new grandchild(ren), did not provide care, co-resided | -0.06 (0.18) |
| Had new grandchild(ren), provided care and co-resided | 0.06 (0.09) |
| Number of observations | 7,746 |

Notes: Data source: CHARLS 2011, 2013, 2015. Beta coefficients are presented. Standard errors are in parenthesis. All models control for age, age square, gender, *hukou* status, educational level, marital status, employment status and log household annual total income.

* p<0.05

Table A8. Transitioning to grandparenthood and ADL limitation among potential first-time grandparents (n=3,449) using a detailed category of grandparent-grandchild contact, with interaction with older adults’ gender (female = ref), LpmFE

| Contact with the new-born | ≥ 1 ADL limitation |
| --- | --- |
| No grandchild at all (ref) |  |
| Had new grandchild(ren), neither provided care nor co-resided | 0.03 (0.02) |
| Had new grandchild(ren), provided care, did not co-reside | 0.04* (0.02) |
| Had new grandchild(ren), did not provide care, co-resided | 0.11 (0.09) |
| Had new grandchild(ren), provided care and co-resided | 0.12** (0.05) |
| Had new grandchild(ren), neither provided care nor co-resided  × Male | -0.03 (0.02) |
| Had new grandchild(ren), provided care, did not co-reside  × Male | -0.09*** (0.02) |
| Had new grandchild(ren), did not provide care, co-resided  × Male | -0.02 (0.13) |
| Had new grandchild(ren), provided care and co-resided  × Male | -0.10 (0.07) |
| Number of observations | 8,531 |

Notes: Data source: CHARLS 2011, 2013, 2015. Beta coefficients are presented. Standard errors are in parenthesis. All models control for age, age square, *hukou* status, educational level, marital status, employment status and log household annual total income.

***p<0.001, ** p<0.01, * p<0.05
